# Correction: Niederhauser et al. Anti-SARS-CoV-2 Antibody Development over Four Years in Blood Donors. *Viruses* 2025, *17*, 1292

**DOI:** 10.3390/v17111447

**Published:** 2025-10-31

**Authors:** Christoph Niederhauser, Nadja Widmer, Caroline Tinguely, Franziska Suter Riniker, Stefano Fontana, Andreas Buser, Sophie Waldvogel, Jutta Thierbach, Max Züger, Peter Gowland

**Affiliations:** 1 Interregional Blood Transfusion SRC, 3008 Bern, Switzerland; nadja.widmer@itransfusion.ch (N.W.); caroline.tinguely@itransfusion.ch (C.T.); stefano.fontana@itransfusion.ch (S.F.); peter.gowland@sunrise.ch (P.G.); 2 Institute of Infectious Diseases, University of Bern, 3001 Bern, Switzerland; franziska.suter@unibe.ch; 3 Faculty of Biological Medicine, Lausanne University Hospital, 1005 Lausanne, Switzerland; 4 Blood Transfusion Service SRC Della Svizzera Italiana, 6900 Lugano, Switzerland; 5 Blood Transfusion Service SRC Beider Basel, 4031 Basel, Switzerland; andreas.buser@usb.ch; 6 Blood Transfusion Service SRC Genève, 1211 Genève, Switzerland; sophie.waldvogelabramowski@hcuge.ch; 7 Blood Transfusion Service SRC Nordostschweiz, 9000 St. Gallen, Switzerland; jutta.thierbach@blutspende-sg.ch; 8 Blood Transfusion Service SRC Thurgau, 8500 Frauenfeld, Switzerland

## Missing Informed Consent Statement

In the original publication [1], the Informed Consent Statement (ICS) was not included in the Back Matter section in the standard format required by the journal. All experiments in the study were conducted in full compliance with the relevant ethical standards, and the corresponding ethics approvals were obtained prior to the study. The corrected statement is provided below. The statement has now been added as follows:

**Informed Consent Statement:** Informed consent was obtained from all subjects involved in the study.

The authors state that the scientific conclusions are unaffected. This correction was approved by the Academic Editor. The original publication has also been updated.
